# 250. Single cell profiling of blood immune cell kinetics in early sepsis reveals progressive decrease in a monocyte substate with immunosuppressive activity

**DOI:** 10.1093/ofid/ofad500.323

**Published:** 2023-11-27

**Authors:** Pierre Ankomah, Alyssa DuBois, Abraham Sonny, Michael Filbin, Marcia B Goldberg, Paul Blainey, Nir Hacohen, Roby P Bhattacharyya

**Affiliations:** Massachusetts General Hospital, Boston, Massachusetts; Broad Institute, Boston, Massachusetts; Massachusetts General Hospital, Boston, Massachusetts; Massachusetts General Hospital, Boston, Massachusetts; Massachusetts General Hospital, Boston, Massachusetts; Broad Institute, Boston, Massachusetts; Massachusetts General Hospital, Boston, Massachusetts; Massachusetts General Hospital, Boston, Massachusetts

## Abstract

**Background:**

Dysregulation of the immune response to bacterial infection is an essential but poorly understood component of sepsis. Kinetic changes in immune cell quantity during sepsis can reveal key elements of immunopathogenesis, yet these data are scant. Single-cell RNA sequencing (scRNA-seq) of peripheral blood mononuclear cells (PBMCs) from sepsis patients in our laboratory has demonstrated a novel monocyte transcriptional state (monocyte substate 1, or MS1) enriched in sepsis. The gene expression profile of MS1 cells is similar to that of myeloid-derived immune suppressor cells (MDSCs), which are key immune regulatory cells that inhibit T cell activation, proliferation, and cytotoxic activity in tumor microenvironments. In this study, we profiled blood samples obtained at multiple time points from patients with sepsis and controls comprising patients with non-infectious critical illness (sterile inflammation) and healthy individuals, to evaluate the kinetics of MS1 and other immune cell transcriptional states.

**Methods:**

PBMCs were obtained from patients with sepsis (n=37), sterile inflammation (n=18) and healthy controls (n=8) at hospital presentation (Day 0), and on Day 1 and Day 3 of their clinical course. We analyzed ∼1500 single cells per sample and identified immune cell states by subclustering within immune cell lineages. Substate abundances were compared between patient phenotypes and across time using Wilcoxon rank sum testing with Benjamini-Hochberg correction.

**Results:**

The fractional abundance of the MS1 substate was higher in sepsis at all time points (Figure 1) and decreased in sepsis patients from Day 0 to Day 3 (padj=0.027). Naïve and memory CD4+T and CD8+T fractions negatively correlated with MS1 fraction at Day 0 for sepsis patients (significant Pearson’s correlations < -0.4).

**Figure 1.**
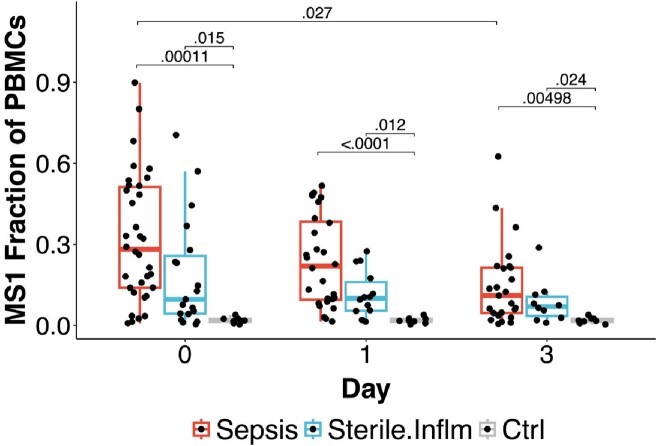
Monocyte substate 1 (MS1) fractional abundance progressively decreases in sepsis patients.

MS1 fraction of total peripheral blood mononuclear cells for each subject on Day 0, 1 and 3 of sampling. Subjects are aggregated by sepsis, sterile inflammation, and healthy control phenotypes; controls are sampled once but plotted at all time points to facilitate comparison. Boxes show the median and inter-quartile range (IQR) for each patient cohort, with whiskers extending to 1.5x the IQR in either direction from the top or bottom quartile. Adjusted p values are shown for inter-phenotype comparisons (two-tailed Wilcoxon rank-sum test with Benjamini-Hochberg correction for testing of multiple states).

**Conclusion:**

This study demonstrates progressively decreasing abundance of monocytic MDSCs in early sepsis. Negative correlations between MS1 and T cell fractions on presentation, but not later, suggest that these monocytes may facilitate systemic immunosuppressive activity very early in sepsis; the subsequent decrease in abundance may limit prolonged immunosuppression and facilitate an adaptive microbicidal response to infection.

**Disclosures:**

**All Authors**: No reported disclosures

